# The Participation of a Malignant Catarrhal Fever Virus and *Mycoplasma bovis* in the Development of Single and Mixed Infections in Beef and Dairy Cattle With Bovine Respiratory Disease

**DOI:** 10.3389/fvets.2021.691448

**Published:** 2021-07-22

**Authors:** Thalita Evani Silva Oliveira, Gabriela Sanches Scuisato, Isadora Fernanda Pelaquim, Cristina Wetzel Cunha, Lucas Santana Cunha, Eduardo Furtado Flores, Lucienne Garcia Pretto-Giordano, Júlio Augusto Naylor Lisbôa, Amauri Alcindo Alfieri, João Paulo Elsen Saut, Paulo Henrique Jorge da Cunha, Selwyn Arlington Headley

**Affiliations:** ^1^Laboratory of Animal Pathology, Department of Veterinary Preventive Medicine, Universidade Estadual de Londrina, Londrina, Brazil; ^2^National Institutes of Science and Technology, Dairy Production Chain (INCT-Leite), Universidade Estadual de Londrina, Londrina, Brazil; ^3^Animal Disease Research Unit, Agricultural Research Service, United States Department of Agriculture, Pullman, WA, United States; ^4^Department of Veterinary Microbiology and Pathology and Paul G. Alan School for Global Animal Health, Washington State University, Pullman, WA, United States; ^5^Department of Statistics, Universidade Estadual de Londrina, Londrina, Brazil; ^6^Department of Preventive Veterinary Medicine, Universidade Federal de Santa Maria, Santa Maria, Brazil; ^7^Laboratory of Veterinary Microbiology and Infectious Diseases, Department of Preventive Veterinary Medicine, Universidade Estadual de Londrina, Londrina, Brazil; ^8^Large Animal Internal Medicine, Department of Veterinary Clinics, Universidade Estadual de Londrina, Londrina, Brazil; ^9^Laboratory of Animal Virology, Department of Preventive Veterinary Medicine, Universidade Estadual de Londrina, Londrina, Brazil; ^10^Large Animal Health Laboratory, Universidade Federal de Uberlândia, Uberlândia, Brazil; ^11^Veterinary Medicine Department, Universidade Federal de Goiás, Goiânia, Brazil; ^12^Programa de Pós-Graduação em Biociência Animal, Universidade de Cuiabá, Cuiabá, Brazil

**Keywords:** diagnostic immunohistochemistry, fibrinoid change, histopathologic patterns, caseonecrotic bronchopneumonia, proliferative vascular alterations, respiratory disease pathogens, ovine gammaherpesvirus 2

## Abstract

The bovine respiratory disease (BRD) complex is a multietiological and multifactorial disease associated with a wide range of viral and bacterial pathogens. This study evaluated the contribution of specific infectious disease agents in the development of BRD in cattle from Brazil and determined if a virus within the malignant catarrhal fever virus (MCFV) group and *Mycoplasma bovis*, acting individually or in conjunction, can be associated with the development of BRD. Formalin-fixed paraffin-embedded pulmonary sections were used in immunohistochemical assays to determine the intralesional presence of six antigens associated with BRD: bovine alphaherpesvirus 1 (BoHV-1), bovine parainfluenza virus 3 (BPIV-3), bovine viral diarrhea virus (BVDV), bovine respiratory syncytial virus (BRSV), MCFV, and *M. bovis*. Pneumonia was diagnosed in 82.7% (120/145) of all cattle evaluated. Interstitial pneumonia (60%, 72/120) and suppurative bronchopneumonia (25.8%, 31/120) were the most frequent patterns of pneumonia identified. Intralesional antigens of MCFV (53.3%, 64/120) were the most frequently associated with BRD, followed by *M. bovis* (47.5%, 57/120), BVDV (42.5%, 51/120), BoHV-1 (28.3%, 34/120), BRSV (24.2%, 29/120), and BPIV-3 (8.3%, 10/120). Additionally, antigens of BVDV, MCFV, and *M. bovis* were the most frequently identified agents associated with singular and concomitant infections. The MCFV identified during this study is more likely to be ovine gammaherpesvirus 2 (OvHV-2), since OvHV-2 is the only MCFV identified within the geographical region of this study. Interstitial pneumonia with proliferative vascular lesions may be a useful histologic feature to differentiate MCFV-induced pneumonia from other viral pneumonias of cattle. These results demonstrate that MCFV and *M. bovis*, in single or mixed infections, can produce pneumonia in cattle and should therefore be considered as primary agents in the development of BRD.

## Introduction

Bovine respiratory disease (BRD) is a complex multifactorial and multietiological disease entity that is associated with viral and bacterial pathogens coupled with unfavorable management practices and environmental conditions. The principal viral disease pathogens associated with the development of BRD are bovine coronavirus (BCoV), bovine alphaherpesvirus 1 (BoHV-1), bovine parainfluenza virus 3 (BPIV-3), bovine viral diarrhea virus (BVDV), and bovine respiratory syncytial virus (BRSV) (1–4). The major bacterial pathogens of BRD include *Mannheimia haemolytica, Pasteurella multocida, Histophilus somni* (5–8), and *Mycoplasma bovis*. All of these pathogens have been associated with outbreaks of BRD in beef and dairy cattle herds from Brazil (9).

More recently, ovine gammaherpesvirus 2 (OvHV-2), a virus within the malignant catarrhal fever virus (MCFV) complex, was suggested as a possible infectious disease pathogen associated with the development of BRD (10). Additionally, it must be highlighted that although the MCFV complex is known to be composed of nine members (11), only OvHV-2 was associated with malignant catarrhal fever (MCF) in ruminants from Brazil (10, 12). Although spontaneous cases of interstitial pneumonia associated with the amplification of OvHV-2 DNA were previously described in bison (13, 14) and buffaloes (15), the possible participation of an MCFV in the development of BRD is a novel finding.

Viral disease pathogens of BRD may cause primary infections, acting in either single or mixed infections with other pathogens (3, 16, 17). A significant role for BRD-related viruses is their interaction with bacteria (17–19) and mycoplasma (20–22) in the development of bronchopneumonia. Moreover, studies have shown that *M. bovis* was the only pathogen identified in pneumonic lungs, resulting in fatal pneumonia in calves (3, 23, 24) and adult cattle (16).

In Brazil, information relative to the occurrence of BRD is limited when compared with studies from North America (25–28) and Australia (29, 30). Previous studies done in Brazil were designed to investigate a single (31–37) or several agents (2, 16, 38–43) associated with BRD. These studies focused on the molecular identification of agents, with and without the histopathologic diagnosis of the patterns of pulmonary disease. It must be highlighted that associating the pattern of pulmonary disease with the intralesional presence of the agents is fundamental to confirming infection. Recently, we have identified the occurrence of infectious disease pathogens of BRD by using a combination of the histopathologic patterns and the *in situ* identification of the infectious disease pathogen by immunohistochemistry (IHC) and have shown that *M. bovis* may be a potential primary disease agent of pneumonia in cattle (16).

Consequently, the aims of this study were to determine the occurrence of respiratory infectious disease agents in cattle from several geographical regions of Brazil, evaluate the contribution of specific infectious disease agents in the development of BRD, and determine if an MCFV and *M. bovis*, acting individually or in mixed infections, can be associated with the development of BRD.

## Materials and Methods

### Study Design

A cross-sectional study focusing on two units of observation was designed: the first investigated respiratory agents associated with the development of BRD, while the second determined the occurrence of pneumonia. The first was designed to associate the occurrence of pneumonia with specific infectious disease pathogens irrespective of the pattern of pulmonary disease. Subsequently, predetermined patterns of pneumonia were classified and related with specific agent(s) associated with BRD.

### Study Area, Animal Selection, and Histopathologic Analyses

A review of all pulmonary tissues of cattle submitted for histopathologic diagnosis between 2015 and 2018 was done to determine the occurrence of pneumonia. These samples originated from diagnostic laboratories located within the states of São Paulo (Southeastern), Paraná, and Santa Catarina (Southern) Brazil; some of the pulmonary tissues derived from cows from Paraná were part of a larger study that investigated the occurrence of cattle neurological disease (44). Archival records of all animals were retrieved, reviewed, and tabulated to obtain information relative to sex, type of animal (beef, dairy, or mixed), age, and cause of death (natural × slaughter). These data were then associated with the occurrence of pulmonary disease. The age of all cattle was divided into two predetermined categories: calves (cattle up to 15 months old) and adults (animals 16 months or older) (45). Only data relative to the age of cattle with pulmonary disease, irrespective of the patterns of pneumonia, were included in the analysis.

Formalin-fixed paraffin-embedded (FFPE) pulmonary sections were used to produce new histological slides when necessary. All sections were stained by the hematoxylin-and-eosin (H&E) method and reviewed for histopathologic patterns of pulmonary disease as outlined (16); the histopathologic review was done by two veterinary pathologists (TESO and SAH). In addition, new histological slides were made for all tissues containing intralesional pleomorphic organisms; these were colored with the Giemsa histochemical method for the identification of organisms consistent with *M. bovis* and with the Brown–Brenn Gram histochemical stain to differentiate them from other accumulations of Gram-positive or Gram-negative bacteria; both methods were based on previous protocols (46). Giemsa staining was used to identify *M. bovis*, since we have previously suggested that this histochemical stain may efficiently identify these intralesional organisms (16).

Additionally, the pulmonary tissues evaluated were divided into three categories based on the predominant histologic alterations observed: (1) pulmonary tissues with predominantly cellular and vascular alterations (congestion, reversible, and irreversible cellular lesions); (2) interstitial pneumonia, and (3) bronchopneumonia. These categories were then used as inputs to correlate these histologic findings with the intralesional localization of antigens of the evaluated agents identified by IHC.

### IHC Identification of Infectious Disease Agents Associated With BRD

IHC assays were performed on pulmonary sections of each animal to determine the intralesional presence of six antigens potentially associated with the development of BRD: BoHV-1, BRSV, BVDV, BPIV-3, MCFV, and *M. bovis*. Selected FFPE tissue sections of the lungs were prepared on silanized slides with poly-l-lysine 0.1% (Sigma-Aldrich, St. Louis, MO, USA) and submitted to IHC assays designed to identify the antigens of these agents. The IHC assays to detect antigens of BoHV-1, BVDV, BRSV, BPIV-3, and *M. bovis* were previously described (16). MCFV-specific antigens were identified by using the monoclonal antibody 15A (MAb-15A) (12). Positive controls included FFPE tissue sections known to contain antigens of BoHV-1, BVDV, BRSV, BPIV-3, *M. bovis* (16), and OvHV-2 (12). Two negative controls were used: the first consisted of substituting the primary antibodies with their respective diluents, and the second consisted of utilizing the primary antibodies on FFPE tissues with known negative immunoreactivity to the BRD antigens derived from the studies cited above. Positive and negative controls were included in each IHC assay.

### Data Analysis

The association between the biological data of the cattle and the occurrence of pneumonia and the frequencies of infection (absolute and relative) was determined using descriptive statistics. The determination of the comparative distribution of IHC antigens in normal and affected tissues was obtained due to the comparative frequency relative to each agent. Consequently, the frequency of each agent evaluated in a determined histologic element was obtained by calculating the occurrence of positive immunolabeling within the histologic element with the total IHC identification of each disease pathogen. Additionally, the association between the occurrence of pathogens and the number of cattle infected was determined. When appropriated, the association between the presence of pulmonary disease and specific variables was analyzed using the chi-square test, using the free software R 4.0.3 (47); differences were considered significant when the resulting *p*-value was < 0.05.

## Results

### Occurrence of Pneumonia and Biological Data

During the 4-year period, the lungs from 145 cattle submitted for histopathologic diagnosis were reviewed. Pneumonia was diagnosed in 82.7% (120/145) of these lungs, and 25 animals had no histopathologic lesions consistent with pneumonia. No significant differences (*p* > 0.05) were identified between the type of cattle, sex, and age relative to the occurrence of pneumonia (Table 1). The median age of the calves was 2 months (range: 2 days to 13 months), while that of adult animals was 4 years (range: 1 year and 8 months to 10 years).

**Table 1:** Epidemiological data of pulmonary tissue of cattle submitted for histopathologic diagnosis.

**Variable**	**Pulmonary tissue without pneumonia (%)**	**Pulmonary tissue with pneumonia (%)**	**Chi-square test (*p*-value)**
**Sex (*****n*****)**			
Male (19)	6	13	0.406
Female (54)	13	41	
Not reported (72)	13	59	
**Type of cattle (*****n*****)**			
Beef (49)	10	39	0.366
Dairy (34)	5	29	
Mixed (62)	17	45	
**Age (*****n*****)**			
Calves (51)	12	39	0.097
Adult (94)	20	74	
**Form of death (*****n*****)**			
Natural (108)	27	81	0.146
Slaughter (37)	5	32	

### Patterns of Pulmonary Disease and Histologic Features of Pneumonia

Most of the lungs evaluated (82.7%, 120/145) had at least one patterns of pneumonia, and some contained more than one patterns of pulmonary disease (Table 2). Consequently, from the 120 animals with pneumonia evaluated, 139 patterns of pulmonary disease were observed, with animals presenting one (72.5%, 87/120) or several patterns (27.5%, 33/120) of pneumonia. Nevertheless, interstitial pneumonia (60%, 72/120) was the most predominant pattern observed (Table 2), followed by suppurative (25.8%, 31/120), caseonecrotic (8.3%, 10/120), and fibrinosuppurative bronchopneumonia (4.2%, 5/120). A few cows (1.7%, 2/120) had cuffing pneumonia.

**Table 2:** Frequency of pneumonia patterns identified in lungs of affected cattle.

**Patterns of pneumonia**	**# Number of cattle**	**%**
Interstitial pneumonia	72	49.7
Suppurative bronchopneumonia	31	21.4
Caseonecrotic bronchopneumonia	10	6.9
Fibrinosuppurative bronchopneumonia	5	3.4
Cuffing pneumonia	2	1.4
Total	120	100

**Figure 1:**
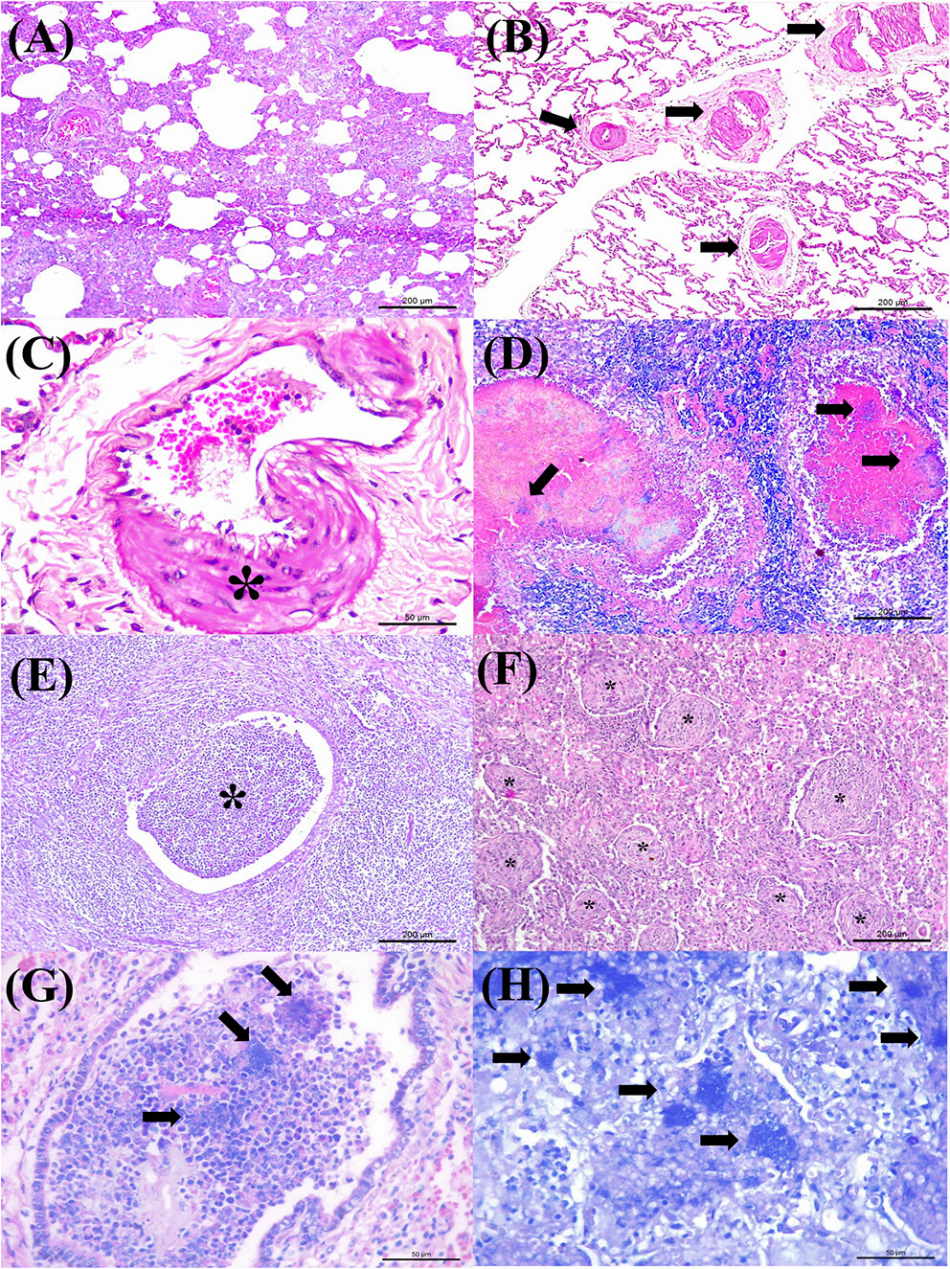
Histopathological findings observed in the lungs of cattle with bovine respiratory disease associated with intralesional antigens of MCFV **(A–C)** and *M. bovis* **(D–H)**. **(A)** There is interstitial pneumonia, **(B)** containing areas of arterial proliferation (arrows); **(C)** higher magnification demonstrating proliferation of the tunica media of pulmonary arteries (asterisk). **(D)** Typical demonstration of caseonecrotic bronchopneumonia associated with infection by *M. bovis*; observe the well-demarcated, centrally located, foci of necrosis containing a large necrotic hyper-eosinophilic debris and pleomorphic bacteria (arrows). **(E)** There is a neutrophilic exudate within a terminal bronchiole (asterisk) in a cow with suppurative bronchopneumonia. **(F)** Observe areas of obliterative bronchiolitis (asterisk). **(G)** There are intralesional bacterial aggregates positive for *M. bovis* (arrows). **(H)** Closer view demonstrating intralesional mollicutes (arrows). Hematoxylin and eosin **(A–F)** and Giemsa stain **(G,H)**. Bar: **(A,B,D–F)** 200 μm; **(C,G,H)** 50 μm.

Antigens of all agents investigated were identified in all three categories of pulmonary lesions evaluated (Table 3). However, antigens of MCFV were more frequently associated with all categories (53.3%, 64/120), including pulmonary tissues with interstitial pneumonia (28.3%, 34/120; Table 3) and vascular disease resulting in arterial proliferation (Figures 1A–C). Other agents frequently associated with the development of interstitial pneumonia were BVDV (25%, 30/120) and BoHV-1 (17.5%, 21/120; Table 3). As expected, antigens of *M. bovis* were more frequently (25%, 30/120) associated with the development of bronchopneumonia (Table 3 and Figure 1D) and suppurative infiltrate predominantly in the terminal and respiratory bronchioles (Figure 1E) and frequently associated with obliterative bronchiolitis (Figure 1F). Intralesional, Giemsa-positive (Figures 1G,H), and bacterial accumulations were observed only in cases of bronchopneumonia (64.6%, 31/48; Table 2). Gram-positive or Gram-negative bacteria were not detected by the modified Brown–Brenn stain.

**Table 3:** Occurrence of respiratory pathogens, according to the pulmonary categories evaluated (*n* = 145)^a^.

**Disease pathogens**	**Cellular/vascular alterations [n (%)]**	**Interstitial pneumonia [n (%)]**	**Bronchopneumonia [n (%)]**	**Total [n (%)]**
Malignant catarrhal fever virus	11 (7.6)	34 (23.4)	19 (13.1)	64 (44.1)
*Mycoplasma bovis*	13 (9)	14 (9.7)	30 (20.7)	57 (39.3)
Bovine viral diarrhea virus	8 (5.5)	30 (20.7)	13 (9)	51 (35.2)
Bovine alphaherpesvirus-1	3 (2.1)	21 (14.5)	10 (6.9)	34 (23.4)
Bovine respiratory syncytial virus	6 (4.1)	17 (11.7)	6 (4.1)	29 (20)
Bovine parainfluenza virus-3	4 (2.8)	4 (2.8)	2 (1.4)	10 (6.9)

a *Based on the occurrence of singular and simultaneous pathogens*.

### IHC Identification of Infectious Disease Antigens in Cattle With BRD

**Figure 2:**
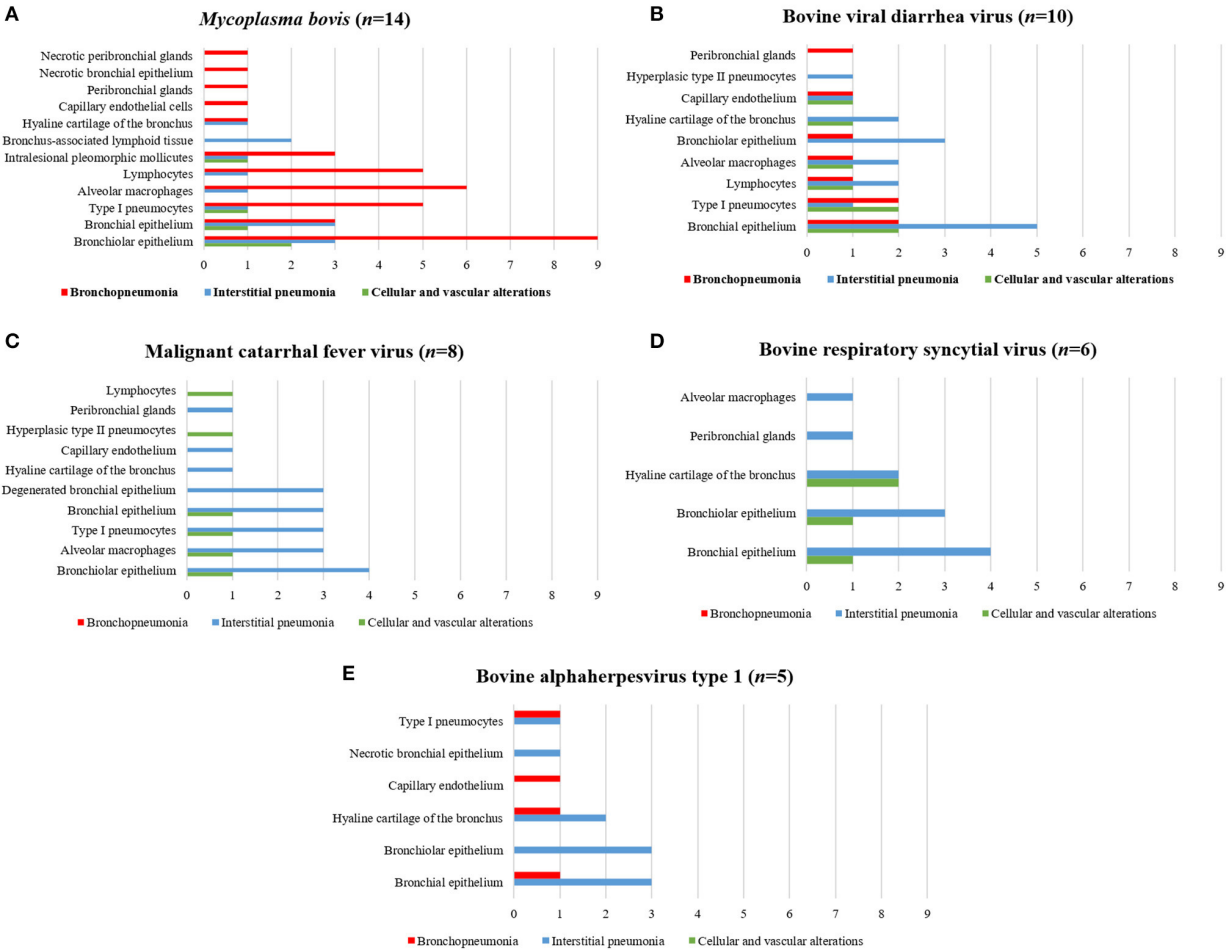
Comparative distribution of the IHC identification of antigens of single infectious disease pathogens within the lungs of cattle with BRD due to **(A)** *M. bovis*, **(B)** BVDV, **(C)** MCFV, **(D)** BRSV, and **(E)** BoHV-1. Bar: green, cellular and vascular alterations; blue, interstitial pneumonia; and red, bronchopneumonia.

The associations between intralesional antigens of infectious disease pathogens of BRD with the specific histologic element of the lung are summarized in Figure 2. When IHC results were associated with a single pathogen (Table 4), *M. bovis* (31.8%, 14/44) was the most frequently identified agent, followed by BVDV (22.7%, 10/44), MCFV (18.2%, 8/44), BRSV (13.6%, 6/44), BoHV-1 (11.4%, 5/44), and BPIV-3 (2.3%, 1/44).

**Table 4:** Distribution of the IHC identification of intralesional antigens in the development of singular and concomitant infections in the lungs of cattle with pneumonia (*n* = 120).

**Summary of singular (*****n*** **=** **44) and dual (*****n*** **=** **40) infections**						
**Agents**	**MCFV**	***M. bovis***	**BVDV**	**BoHV-1**	**BRSV**	**BPIV-3**
MCFV	8	10	12	3	3	0
*M. bovis*	–	14	2	2	2	2
BVDV	–	–	10	0	2	0
BoHV-1	–	–	–	5	1	1
BRSV	–	–	–	–	6	0
BPIV-3	–	–	–	–	–	1
**Association of intralesional antigens**						**Number of cattle**
**Triple infections**	
BoHV-1, BPIV-3, BRSV						1
BoHV-1, BRSV, *M. bovis*						2
BoHV-1, BVDV, *M. bovis*						3
BoHV-1, BVDV, MCFV						6
BoHV-1, *M. bovis*, MCFV						1
BPIV-3, BRSV, *M. bovis*						1
BRSV, BVDV, MCFV						1
BRSV, *M. bovis*, MCFV						3
BVDV, *M. bovis*, MCFV						7
Total						25
**Quadruple infections**	
BoHV-1, BPIV-3, BVDV, *M. bovis*						1
BoHV-1, BRSV, BVDV, MCFV						3
BoHV-1, BRSV, *M. bovis*, MCFV						1
BoHV-1, BVDV, *M. bovis*, MCFV						2
BPIV-3, BRSV, *M. bovis*, MCFV						1
BRSV, BVDV, *M. bovis*, MCFV						1
Total						9
**Quintuple infections**	
BoHV-1, BPIV-3, BRSV, *M. bovis*, MCFV						1
BoHV-1, BPIV-3, BVDV, *M. bovis*, MCFV						1
Total						2

*BoHV-1, bovine alphaherpesvirus type 1; BPIV-3, bovine parainfluenza virus type 3; BRSV, bovine respiratory syncytial virus; BVDV, bovine viral diarrhea virus; M. bovis, Mycoplasma bovis; MCFV, malignant catarrhal fever viruses*.

**Figure 3:**
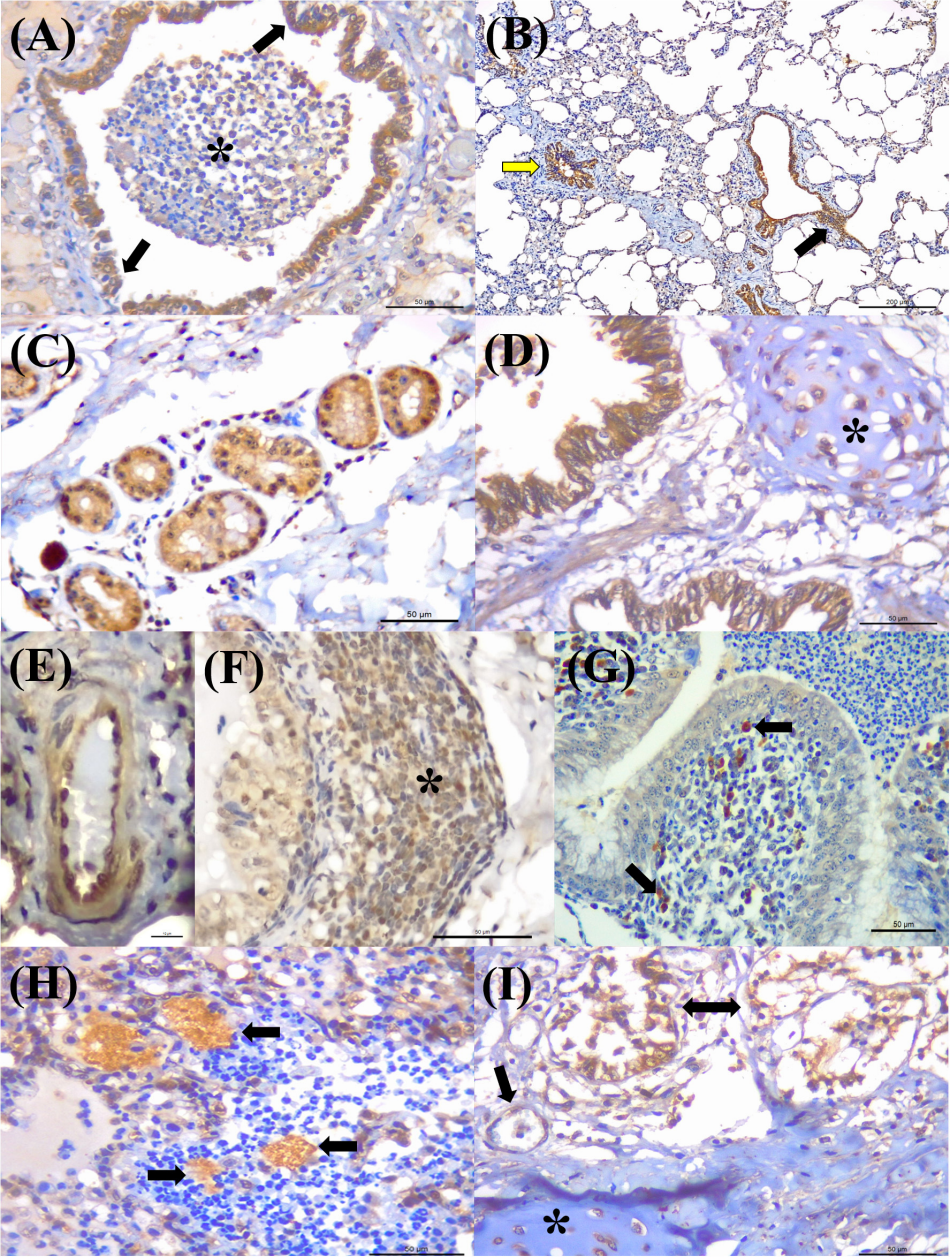
Immunohistochemical detection *Mycoplasma bovis* antigens in cattle with bovine respiratory disease. **(A)** There is positive intracytoplasmic immunoreactivity to *M. bovis* antigens in the normal bronchiolar epithelium (arrows) of a cow with bronchopneumonia (asterisk). **(B)** Observe positive immunoreactivity at the respiratory (yellow arrow) and terminal bronchiole (black arrow) of a cow with interstitial pneumonia. **(C)** There is positive reactivity at the mixed peribronchial glands. **(D)** Observe positive immunoreactivity within chondrocytes of the hyaline cartilage (asterisk), **(E)** endothelium cells, **(F)** lymphocytes (asterisk), **(G)** macrophages, (arrow), and bronchus-associated lymphoid tissue. **(H)** There is positive immunolabeling within accumulations of pleomorphic organism (arrows) in the lung with suppurative bronchopneumonia. **(I)** Observe positive immunolabeling in necrotic peribronchial glands (two-headed arrow), normal endothelium cells (arrow), and chondrocytes of the hyaline cartilage (asterisk). Immunoperoxidase counterstained with hematoxylin. Bar: **(A,C,D,F–I)** 50 μm; **(B)** 200 μm; **(E)** 10 μm.

Positive immunoreactivity to *M. bovis* antigens was widely distributed within the lung during this study (Figure 2A). These include the epithelial cells of the bronchiole (Figures 3A,B) and bronchus, alveolar macrophages, type I pneumocytes, mixed peribronchial glands (Figure 3C), chondrocytes of the bronchial hyaline cartilage (Figure 3D), and endothelium (Figure 3E). Furthermore, *M. bovis* was the only agent associated with positive immunolabeling on BALT lymphoid tissue (Figures 3F,G) and intralesional pleomorphic mollicutes (Figure 3H). Antigens of *M. bovis* were also identified within necrotic peribronchial glands (Figure 3I) and necrotic bronchial epithelial cells.

**Figure 4:**
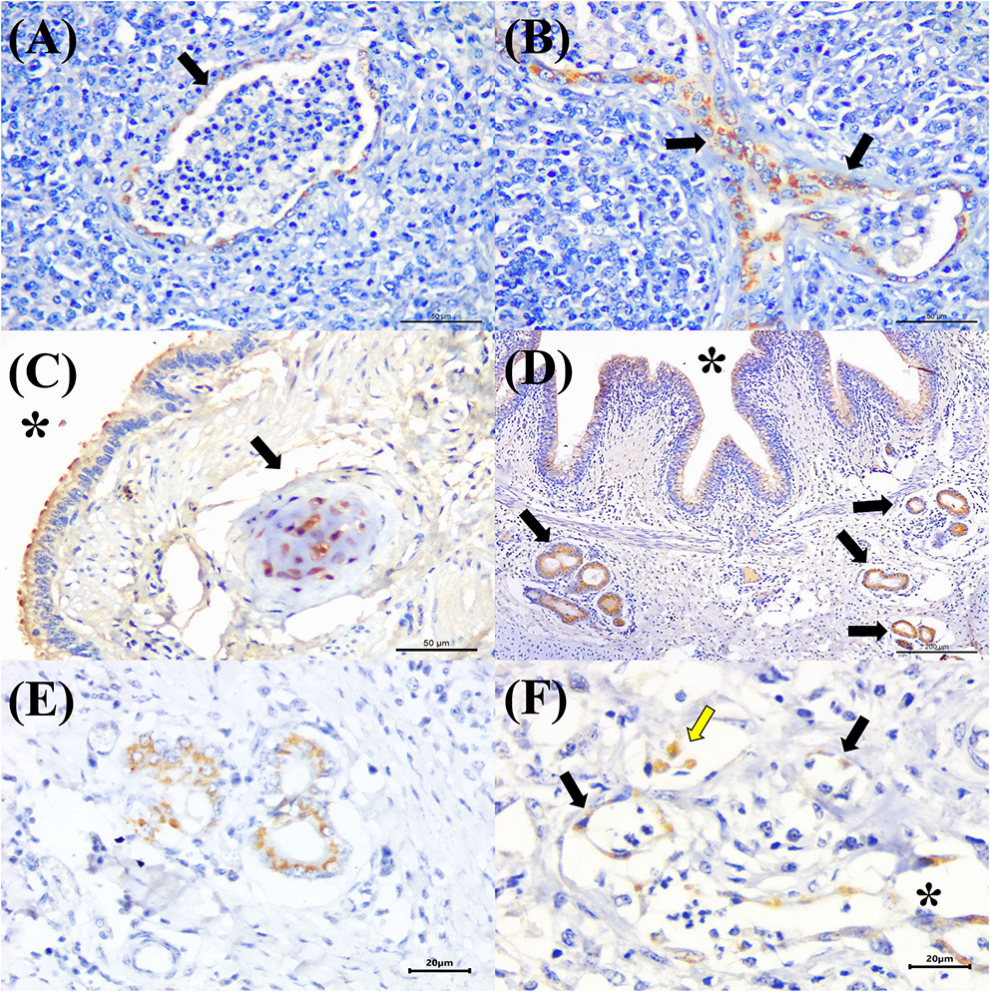
Immunohistochemical detection of BVDV antigens in cattle with bovine respiratory disease. There is positive immunoreactivity (arrows) to BVDV in the epithelial cells of the **(A)** terminal and **(B)** respiratory bronchiole of a cow with bronchopneumonia. **(C)** Observe positive immunolabeling at chondrocytes of the hyaline cartilage (asterisk), epithelial cells of the bronchus (arrow), **(D)** mixed peribronchial glands (arrows), and bronchial epithelial cells (asterisk); **(E)** closer view showing positive immunoreactivity within epithelial cells of the mixed peribronchial glands. **(F)** BVDV antigens within alveolar macrophages (yellow arrow), the endothelia of a capillary (black arrows), and venule (asterisk) of the lung. Immunoperoxidase counterstained with hematoxylin. Bar: **(A–C)** 50 μm; **(D)** 200 μm; **(E,F)** 20 μm.

BVDV antigens were observed in the three categories studied (Figure 2B). Immunoreactivity was observed within the epithelial cells of the bronchus and bronchiole (Figures 4A,B), type I pneumocytes, alveolar lymphocytes and macrophages, chondrocytes of the bronchial hyaline cartilage (Figure 4C), mixed peribronchial glands (Figures 4D,E), endothelium cells (Figure 4F), and hyperplastic type II pneumocytes.

**Figure 5:**
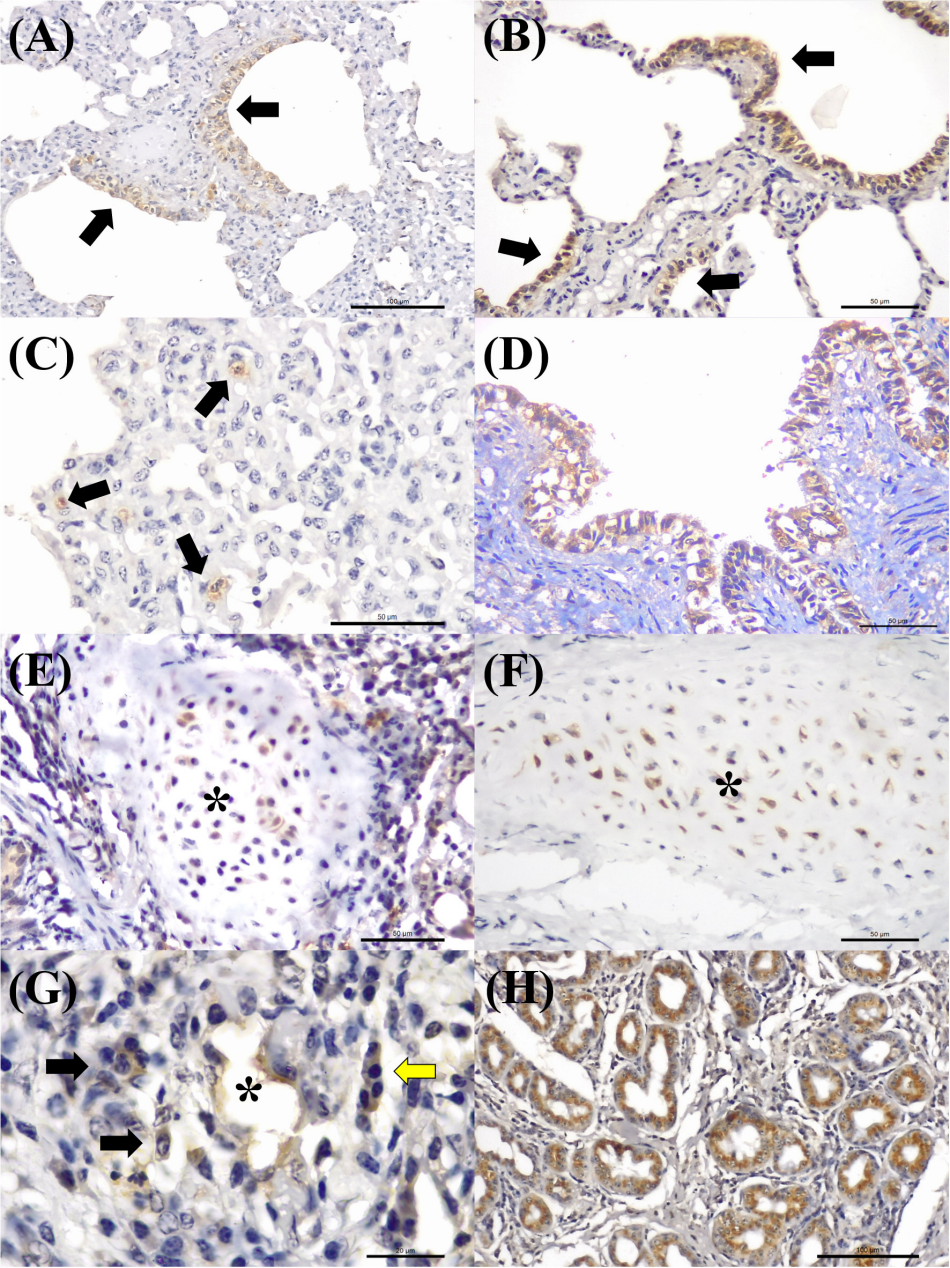
Immunohistochemical demonstration of immunoreactivity to MCFV in cattle with bovine respiratory disease. There is positive intracytoplasmic immunoreactivity to antigens of MCFV within the cytoplasm of epithelial cells of the **(A,B)** terminal bronchiole and the patchy immunoreactivity within **(C)** alveolar epithelium (arrows) in a case of interstitial pneumonia. Observe intracytoplasmic immunoreactivity to antigens of MCFV within **(D)** degenerated bronchial epithelial cells, **(E,F)** chondrocytes of the hyaline cartilage (asterisk), **(G)** endothelium cells of a pulmonary venule (asterisk), macrophages (black arrows), lymphocytes (yellow arrow), and **(H)** epithelial cells of the mixed peribronchial glands. Immunoperoxidase counterstained with hematoxylin. Bar: **(A,H)** 100 μm; **(B–F)** 50 μm; and **(G)** 20 μm.

MCFV-positive intracytoplasmic immunoreactivity (Figure 2C) was identified primarily within bronchiolar epithelial cells (Figures 5A,B), alveolar macrophages (Figure 5C), type I pneumocytes, degenerated bronchial epithelial cells (Figure 5D), chondrocytes of the bronchial hyaline cartilage (Figures 5E,F), endothelial cells of pulmonary venule (Figure 5G), hyperplasic type II pneumocytes, mixed peribronchial glands (Figure 5H), and alveolar lymphocytes. Positive immunoreactivity to antigens of MCFV was restricted to the pulmonary lesions within the categories classified as cellular and vascular alterations and interstitial pneumonia, without being observed in tissues diagnosed as bronchopneumonia. Additionally, immunoreactivity to MCFV antigens was patchy within the pneumocytes of cows with interstitial pneumonia and was more predominant in type I relative to type II cells.

**Figure 6:**
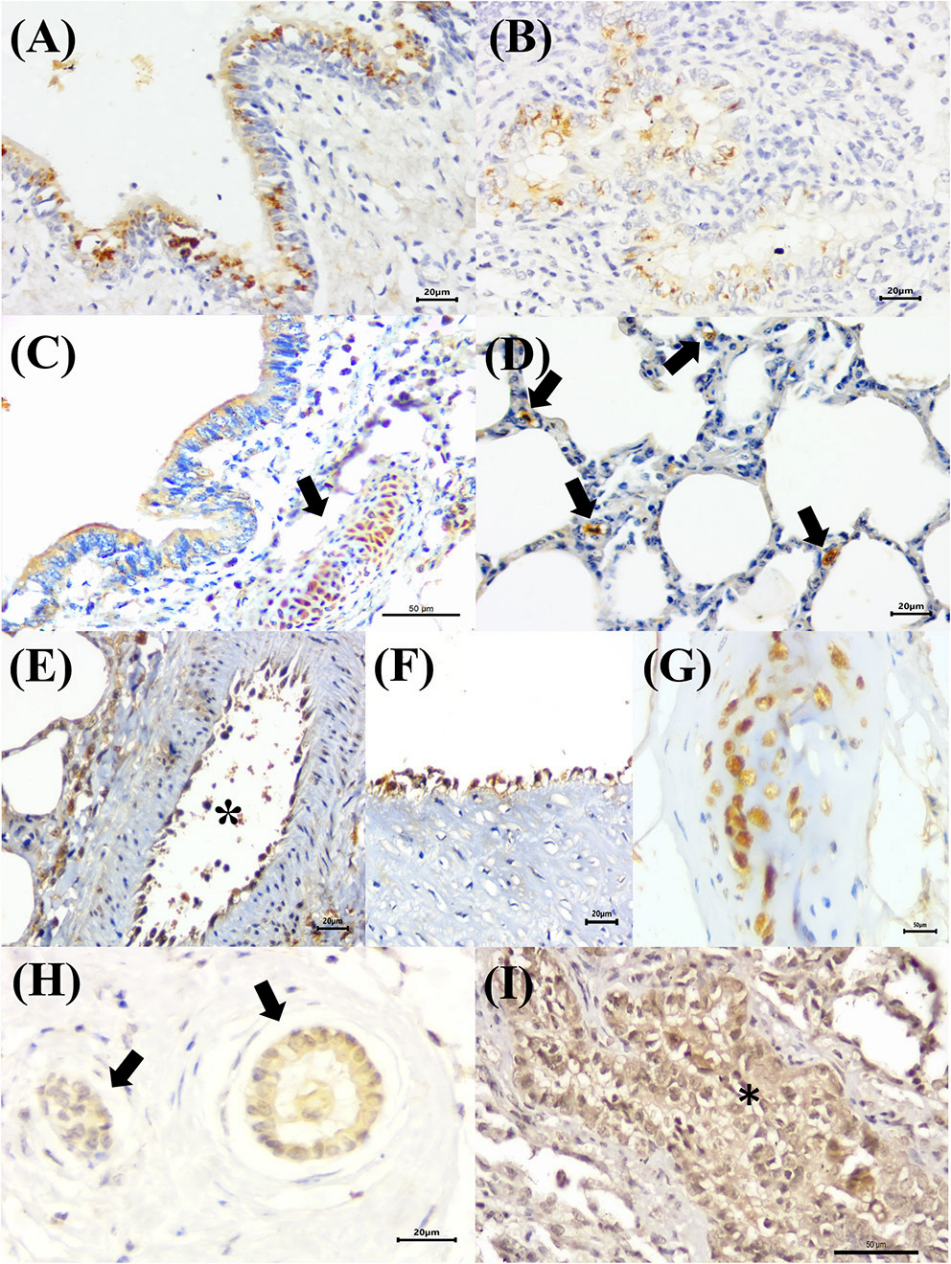
Immunohistochemical identification of BRSV, BoHV-1, and BPIV-3 antigens in singular infections of cattle with bovine respiratory disease. **(A)** There is positive intracytoplasmic immunoreactivity to BRSV antigens within bronchial and **(B)** bronchiolar epithelial cells, **(C)** chondrocytes of the hyaline cartilage (arrow), and **(D)** alveolar macrophages (arrows). **(E,F)** Observe positive intracytoplasmic immunolabeling for BoHV-1 antigens within endothelial cells [**(E)**, asterisk] and **(G)** chondrocytes of the hyaline cartilage. **(H)** There is positive intracytoplasmic immunoreactivity to BPIV-3 antigens within epithelial cells of the normal (arrows) and **(I)** vacuolized bronchiolar cells (asterisk). Immunoperoxidase counterstained with hematoxylin. Bar: **(A,B,D–F,H)** 20 μm; **(C,G,I)** 50 μm.

Immunoreactivity to BRSV (Figure 2D), with positive intracytoplasmic immunoreactivity, occurred within bronchial and bronchiolar epithelial cells (Figures 6A,B), chondrocytes of the bronchial hyaline cartilage (Figure 6C), mixed peribronchial glands, and alveolar macrophages (Figure 6D). Similar to MCFV, BRSV antigens were observed only in the categories classified as circulatory changes and interstitial pneumonia.

BoHV-1 antigens (Figure 2E) were observed only in cases of interstitial pneumonia and bronchopneumonia, with intracytoplasmic immunoreactivity within bronchial and bronchiolar epithelial cells, pulmonary endothelial cells (Figures 6E,F), chondrocytes of the bronchial hyaline cartilage (Figure 6G), necrotic bronchial epithelial cells, and type I pneumocytes.

BPIV-3 antigens were observed only in one animal with circulatory changes and revealed cytoplasmic immunoreactivity within the cells of the normal (Figure 6H) and degenerated (Figure 6I) bronchiolar epithelia.

**Figure 7:**
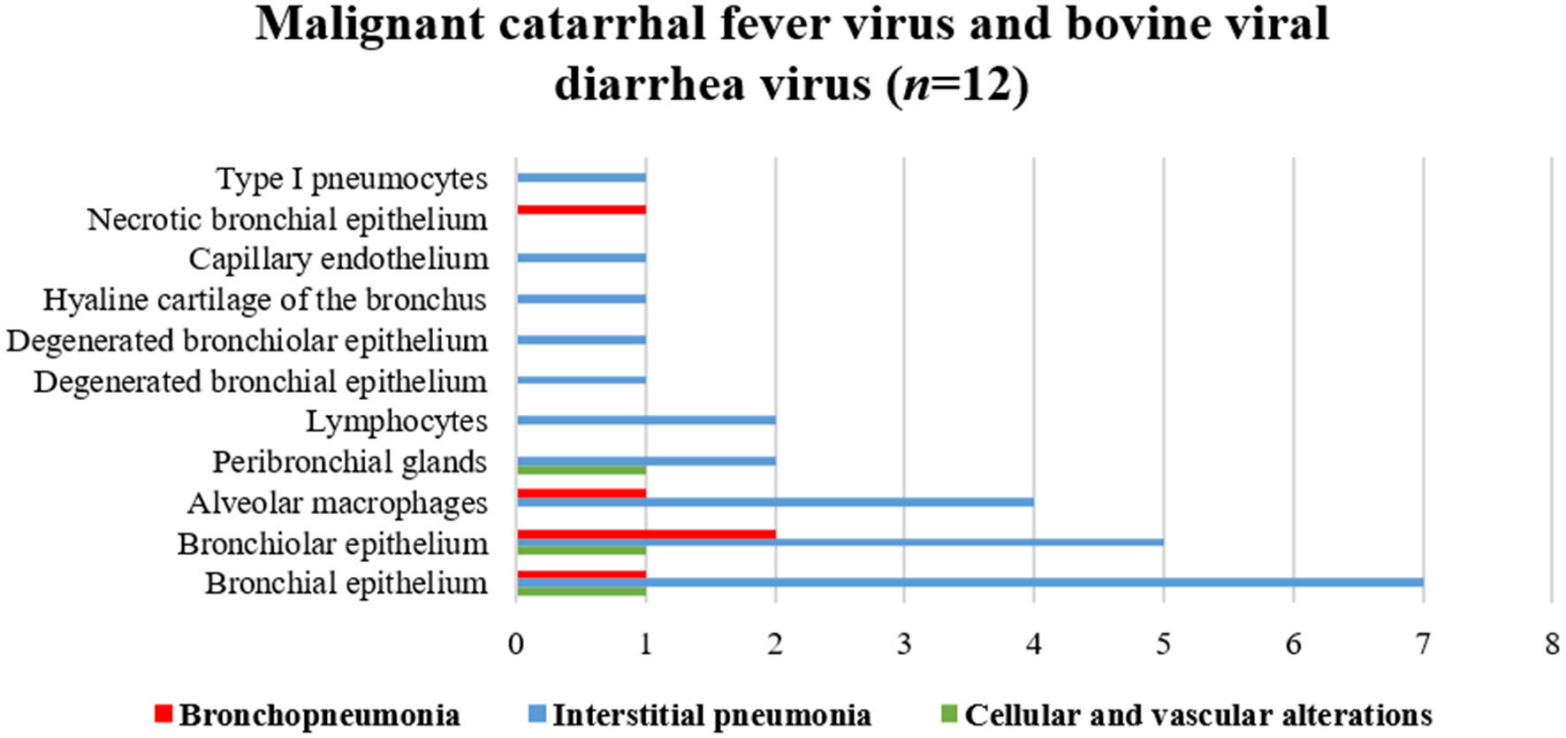
Comparative demonstration of positive immunolabeling for MCFV and BVDV antigens within histologic elements of lungs of cattle with BRD.

**Figure 8:**
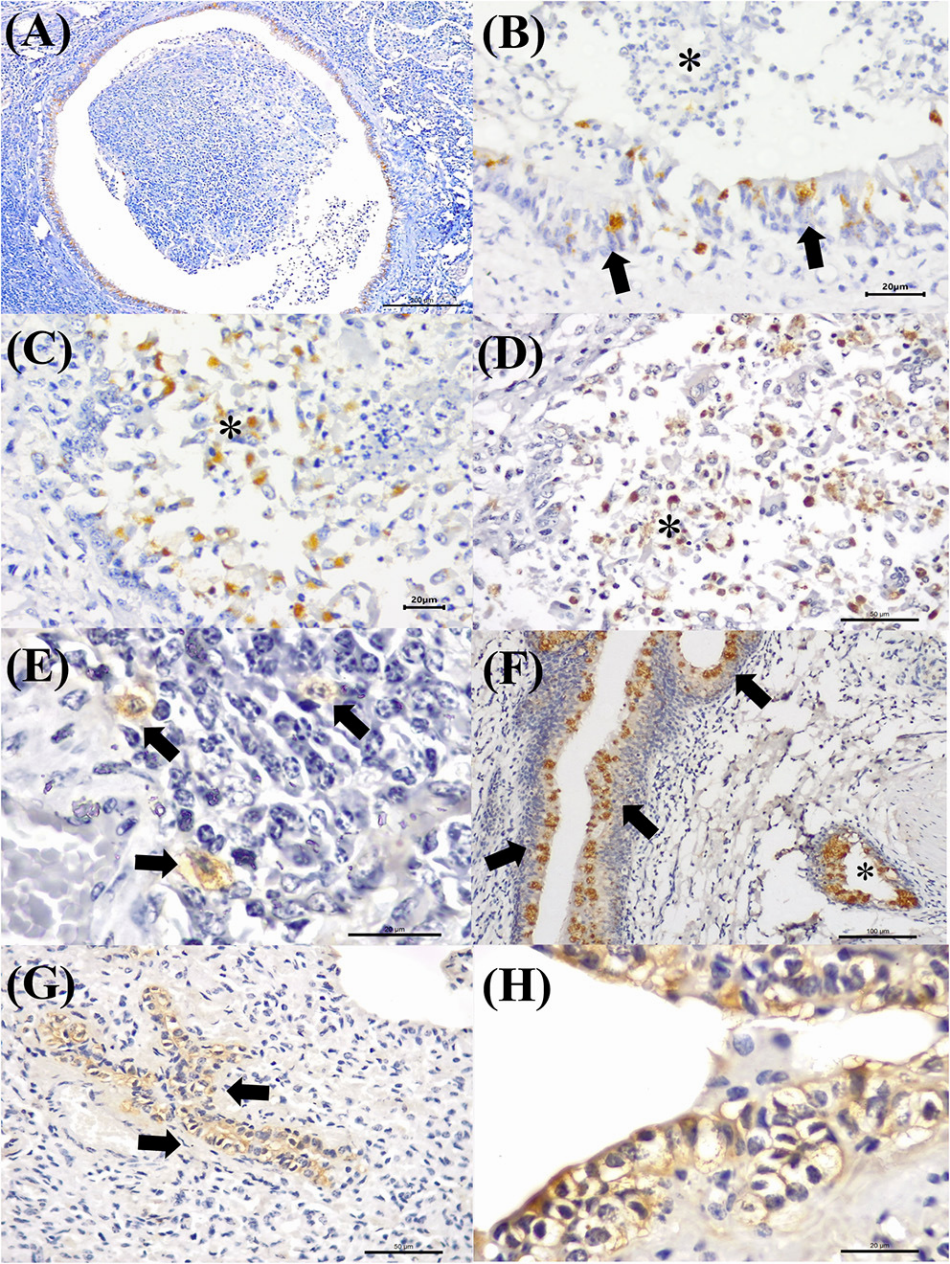
Immunohistochemical identification of BVDV and MCFV antigens in cattle with bronchopneumonia with BRD. **(A)** There is positive intracytoplasmic immunoreactivity to BVDV in the bronchial cells of an ectatic bronchiole due to bronchiectasis, **(B)** closer view of epithelial cells (arrows) and negative immunoreactivity in neutrophils (asterisk). **(C,D)** Immunoreactivity to MCFV antigens within necrotic bronchiolar epithelial cells (asterisk), **(E)** alveolar macrophages (arrows), **(F)** goblet cells (arrows), normal cells (asterisk), and **(G)** degenerated bronchiolar epithelial cells (arrows); **(H)** closer view of the degenerated bronchiolar epithelial cells. Immunoperoxidase counterstained with hematoxylin. Bar: **(A)** 200 μm; **(B,C,E,H)** 20 μm; **(D,G)** 50 μm; **(F)** 100 μm.

Figure 7 illustrates an interesting feature identified in mixed infections due to BVDV and MCFV in 12 animals. Concomitant infections were observed predominantly in bronchopneumonia (16.7%, 2/12) and interstitial pneumonia (75%, 9/12) and were also associated with the development of cellular and vascular alterations (8.3%, 1/12). More interestingly, with simultaneous immunolabeling in bronchopneumonia, antigens of both viruses were identified within bronchial and bronchiole epithelia (Figures 8A,B), necrotic bronchiolar cells (Figures 8C,D), and alveolar macrophages (Figure 8E). Additionally, there was positive immunoreactivity to MCFV antigens within goblet cells (Figure 8F), chondrocytes of the bronchial hyaline cartilage, pulmonary endothelia, mixed peribronchial glands, alveolar lymphocytic infiltrates, and degenerated bronchial epithelia (Figures 8G,H).

### Frequency and Relationship Between the Occurrence of Infectious Disease Pathogens and Pulmonary Disease

A high occurrence of respiratory disease pathogens was observed in the pulmonary tissues evaluated, with antigens of more than one respiratory disease pathogen detected in 82.7% (120/145) of these, while 36.7% (44/120) of the pulmonary tissues contained only one pathogen associated with the development of BRD. When the occurrence of single infections was evaluated (Table 4), *M. bovis* antigens were the most frequently identified (31.8%, 14/44), followed by BVDV (22.7%, 10/44), MCFV (18.2%, 8/44), BRSV (13.6%, 6/44), BoHV-1 (11.4%, 5/44), and BPIV-3 (2.3%, 1/44). However, antigens of infectious disease agents were not identified in 17.2% (25/145) of the pulmonary tissues evaluated. Antigens of MCFV and *M. bovis* (Table 4) were associated with singular (18.2%, 8/44; 22.7%, 10/44), dual (70%, 28/40; 45%, 18/40), triple (76%, 19/25; 68%, 17/25), quadruple (88.9%, 8/9; 66.7%, 6/9), and quintuple (100%, 2/2; 100%, 2/2) infections during this study.

Two respiratory disease agents were simultaneously identified within the lungs of 40 cows (Table 4), resulting in mixed infections. Antigens of MCFV were the most frequently observed to be associated with BVDV (42.9%, 12/28), followed by *M. bovis* (35.7%, 10/28), BRSV (10.7%, 3/28), and BoHV-1 (10.7%, 3/28). Antigens of BVDV were identified in more than half (53.9%, 41/76; Table 4) of these cases, while mixed infections associated with BVDV predominantly included MCFV (40.8%, 31/76) and *M. bovis* (22.4%, 17/76). Additionally, dual infections were more frequently associated with intralesional antigens of MCFV (*n* = 28; Table 4), being predominantly associated with antigens of BVDV (42.8%, 12/28) and *M. bovis* (35.7%, 10/28).

In animals infected with a single agent, infection of normal bronchiolar epithelial cells was observed in all *M. bovis*-infected cows (100%, 14/14), followed by BRSV (83.3%, 5/6), MCFV (50%, 4/8), BVDV (40%, 4/10), and BoHV-1 (40%, 2/5). Few pulmonary tissues had positive immunoreactivity at the capillary endothelium, being observed for BVDV (30%, 3/10), BoHV-1 (20%, 1/5), MCFV (12.5%, 1/8), and *M. bovis* (7.1%, 1/14).

Positive immunolabeling at the hyaline cartilage of the bronchus was identified in the pulmonary tissues of 27.3% (12/44) cows; BRSV (66.7%, 4/6) antigens were more frequently identified within chondrocytes of the hyaline cartilage of the bronchus, followed by BoHV-1 (40%, 2/5), BVDV (30%, 3/10), *M. bovis* (14.3%, 2/14), and MCFV (12.5%, 1/8); antigens of BPIV-3 were not observed within chondrocytes. A few cattle had positive immunolabeling at normal peribronchial glands for antigens of BRSV (16.7%, 1/6), MCFV (12.5%, 1/8), and *M. bovis* (7.1%, 1/14). Positive immunoreactivity within lymphocytes was observed in 11 cows and was associated with antigens of *M. bovis* (54.5%, 6/11), BVDV (36.4%, 4/11), and MCFV (9.1%, 1/11); antigens of BRSV and BPIV-3 were not observed within lymphocytes. Intralesional pleomorphic organisms, stained by Giemsa, were identified in 35.7% (5/14; Figure 3H) of cows that contained antigens of *M. bovis*.

The most frequent infectious disease pathogen (Table 3) identified in association with BRD was MCFV (53.3%, 64/120), followed by *M. bovis* (47.5%, 57/120), BVDV (42.5%, 51/120), BoHV-1 (28.3%, 34/120), BRSV (24.2%, 29/120), and BPIV-3 (8.3%, 10/120). Furthermore, singular (36.7%, 44/120), dual (33.3%, 40/120), triple (20.8%, 25/120), quadruple (7.5%, 9/120), and quintuple (1.7%, 2/120) infections were identified (Table 4).

Necrosis was observed at the epithelial cells and peribronchial glands in the lungs of 6.8% (3/44) cows. Of these cases, necrosis affecting bronchial epithelia was associated with antigens of BoHV-1 (20%, 1/5) and *M. bovis* (7.1%, 1/14), with *M. bovis* antigens being observed in 7.1% (1/14) of the necrotic peribronchial glands.

Hyperplastic lesions were identified in few cows (9.1%, 4/44); in two of these, there was bronchus-associated lymphoid tissue (BALT) hyperplasia associated with positive immunoreactivity to *M. bovis* antigens. Hyperplasia of type II pneumonocytes was observed in two (4.5%, 2/44) cows; with positive immunoreactivity to MCFV (2.3%, 1/44) and BVDV (2.3%, 1/44).

## Discussion

The results of this study demonstrated the multietiological nature of BRD, in which 63.3% (76/120) of the lungs of cattle evaluated were infected by two or more infectious disease agents. The IHC identification of infectious disease pathogens on FFPE tissues is a sensitive method to detect intralesional antigens and was previously used to identify BVDV, BoHV-1, BRSV, *M. bovis* (3, 16), BPIV-3 (16, 48), and MCFV (12) antigens in tissues of BRD-affected cattle. Additionally, the *in situ* detection of intralesional tissue antigens is an excellent method for retrospective studies using archival samples. Moreover, this diagnostic method is preferred over molecular testing, to confirm disease association, since the intralesional identification of disease pathogens within affected tissue clearly demonstrates the association between infectious disease agents and histologic alteration and/or pattern of disease (49, 50).

In a previous study by our group in cattle with neurological manifestation of MCF associated with OvHV-2 but without the classic manifestations of MCF (12), positive immunoreactivity was not observed within the pulmonary tissues available for evaluation. Similarly, in the current study, the cows investigated did not demonstrate the typical clinical manifestations of MCF and were therefore without a clinical diagnosis of MCF. The pulmonary disease associated with MCFV antigens identified during this study can be classified as subacute to chronic interstitial pneumonia due to the accumulated lymphocytes and macrophages (51). Moreover, OvHV-2 is known to produce chronic disease in cattle characterized by proliferating arterial lesions (12, 52, 53); proliferative vascular lesions were observed in the lungs of cattle with interstitial pneumonia associated with intralesional antigens of MCFV and represented 28.3% (34/120) of all interstitial pneumonias identified during this study. These findings may suggest that MCFV produces interstitial pneumonia with vascular proliferating lesions as the prominent histologic feature, which may be useful to distinguish MCFV-induced interstitial pneumonia from other viral pneumonias of cattle. Another interesting feature during this study was the patchy immunoreactivity of pneumocytes in interstitial pneumonia associated with MCFV antigens; similar findings were described in pigs experimentally infected with OvHV-2 (54). Additionally, experimental studies done in sheep (55, 56) and pigs (54) have suggested that the interstitial pneumonia induced by OvHV-2 results in lytic replication predominantly within type II pneumocytes (55). In the present study, intralesional MCFV antigens were observed within hyperplastic type II pneumocytes and degenerated and necrotic bronchiolar and bronchial epithelia; these findings herein described corroborate those of experimental studies (54, 56). Consequently, this pathogen may be an innocent bystander, or a primary infectious disease agent, acting individually or in association with other pathogens toward the development of BRD in cattle, and should be considered a possible infectious agent associated with the development of BRD. Moreover, an MCFV, more likely OvHV-2, was related with the occurrence of respiratory disease in a calf that was simultaneously infected with BVDV and *Aspergillus fumigatus* (57).

Furthermore, the MAb-15A antibody that detects epitopes of MCF viruses (58) did not react with common viruses of sheep, goat, and cattle (58, 59) and was shown to effectively identify intralesional antigens of OvHV-2 in FFPE of cattle with MCF (12). The IHC findings observed in this study demonstrated the participation of MCFV in the development of respiratory disease in the cows evaluated. Furthermore, positive immunoreactivity for MCFV was observed within epithelial cells of the lungs of cattle with different categories of pulmonary disease, but principally in cases of interstitial pneumonia. In Brazil, only OvHV-2 is known to be associated with the development of MCF in ruminants (10, 12), suggesting that the MCFV identified in these animals was most likely OvHV-2; similar findings were recently described (57, 60). Previous investigations have shown that OvHV-2 induces infiltrative, degenerative (60), and necrotic changes in the urinary bladder (60, 61), kidney cells (60), salivary gland (61), and the gastrointestinal and respiratory systems of cattle (57, 61). Furthermore, a review of all published cases of MCF in Brazil revealed that interstitial pneumonia is a frequently diagnosed histologic alteration described in cattle infected with OvHV-2 (10).

Only *M. bovis* antigens were observed in the lungs of cattle with BALT hyperplasia, suggesting that this lesion can be used as an indicator of *M. bovis*-induced pneumonia in cattle as previously described (62, 63). It should be emphasized that in most cases of BALT hyperplasia, the pulmonary tissue evaluated had more than one histologic pattern of *M. bovis*-induced pulmonary disease, while only in two animals was cuffing pneumonia the only histologic alteration observed. Other histologic patterns of *M. bovis*-induced pneumonia identified were caseonecrotic and suppurative bronchopneumonia; similar results have been described (21, 62, 63). The caseonecrotic bronchopneumonia (also referred to as necrosuppurative bronchopneumonia) is considered the most important diagnostic histologic feature to differentiate *M. bovis*-induced pneumonia from other bacterial pneumonias of cattle (21, 62).

A limitation of this study was the absence of the IHC analyses to identify members of the *Pasteurellaceae* family associated with the development of BRD. Although *P. multocida, M. haemolytica*, and *H. somni* are the most common bacterial pathogens associated with bovine pneumonia (6, 18, 51, 64), these pathogens were not evaluated during this study due to the lack commercial antibodies for IHC. Additionally, attempts to obtain in-house polyclonal antibodies or hyperimmune serum against these bacterial agents were frustrating. Nevertheless, these organisms are commensals of the bovine nasopharynx which, during periods of stress or viral infection, can overwhelm host defense mechanisms establishing infection in the lower respiratory tract and are associated with the development of fibrinosuppurative or suppurative bronchopneumonia, pulmonary abscesses, and necrosis in cattle (6, 49, 51, 64). Consequently, the BALT hyperplasia and caseonecrotic bronchopneumonia associated with intralesional antigens of *M. bovis* can be considered as histologic patterns specific for pulmonary disease by this pathogen as opposed to histologic patterns of pulmonary disease related to *P. multocida, M. haemolytica*, and *H. somni*. We had previously postulated that Giemsa staining may be a cheap and adequate method to identify intralesional *Mycoplasma* organisms (16). Similar results were identified in this study, suggesting that this simple histochemical stain may be used to confirm the presence of this organism, principally in cases with the classical histologic presentation of mycoplasma pneumonia.

In this study, 63.3% (76/120) of the pulmonary infections observed were mixed; tissue antigens of MCFV and *M. bovis* were observed simultaneously in two to five infections within the same pulmonary section. These findings suggest that these two organisms can produce pneumonia acting individually or in association with other pathogens of BRD. BVDV is a well-known immunosuppressive agent of cattle (65–72), which could have favored concomitant infections, including MCFV and *M. bovis*. We have previously discussed the relationship between BVDV and *M. bovis* and the synergism between these two organisms (16). In that study, there were four singular infections (11.4%, 4/35) associated with *M. bovis* (16); in the current study, 11.7% (14/120) of the affected cows were infected with *M. bovis*. These results corroborate previous studies that have identified *M. bovis*, in single infections, as a primary contributor toward the development of BRD (3, 20, 73). However, the same does not hold for MCFV or OvHV-2 as described above, since there are few reports associating these pathogens with BRD.

This study demonstrated high frequencies of infections by MCFV (53.3%, 64/120) and *M. bovis* (47.5%, 57/120) in cattle from geographical locations of Brazil. These elevated occurrences can be related to several conditions, including the absence of a vaccine or specific treatment to effectively control MCFV and the frequently chronic presentation of *M. bovis*-induced pneumonia (21), which results in a late diagnosis. Consequently, farmers must be educated relative to the existence of these diseases, especially concerning the adoption of adequate control and prophylactic measures (74). Another factor that may have contributed to the elevated occurrence of *M. bovis* during this study is the well-established microbial resistance of this organism to common antibiotic therapy (21, 75).

## Conclusion

These results suggest that most cattle evaluated presented some form of pulmonary lesions associated with BRD. The occurrence of interstitial pneumonia was most frequently related to antigens of MCFV and BVDV, while *M. bovis* was frequently associated with caseonecrotic bronchopneumonia. These findings suggest that an MCFV, most likely involving OvHV-2, was associated with the development of pulmonary disease in cattle and should be considered as a primary disease pathogen of BRD, acting innocently, singularly, or in association, primarily with BVDV. Moreover, the concomitant occurrence of MCFV and BVDV within the lungs of cattle with pneumonia suggests a possible synergism between these two infectious agents toward the development of BRD. Furthermore, proliferating vascular lesions in the lung may be an important histologic feature to diagnose MCFV-induced interstitial pneumonia in cattle.

## Data Availability Statement

The raw data supporting the conclusions of this article will be made available by the authors, without undue reservation.

## Ethical Statement

The animal study was reviewed and approved by Ethics Committee of the Universidade Estadual de Londrina (CEUA/UEL; protocol, 835.2019.45). Written informed consent was obtained from the owners for the participation of their animals in this study.

## Author Contributions

TO contributed substantially to the conception and design of the study, drafted the manuscript, and contributed to the analysis and interpretation of all pathological, IHC, and statistical data. TO, GS, IP, and SH contributed to all histopathological evaluations, histochemical and IHC stains, interpretation of the IHC, and analyses. CC, LP-G, and EF contributed to the interpretation of the IHC analyses. TO, LC, JL, and JS contributed to the interpretation of the statistical analysis. JL, GS, PJ, and AA contributed toward the supply of pulmonary tissues for this study. SH coordinated and supervised the execution of the study. All authors have read, critically analyzed, and approved the final draft of this manuscript and have agreed to be accountable for all aspects of the study in ensuring that questions related to the accuracy or integrity of any part of the work are appropriately investigated and resolved.

## Conflict of Interest

The authors declare that the research was conducted in the absence of any commercial or financial relationships that could be construed as a potential conflict of interest.
